# High Performance and Structural Stability of K and Cl Co-Doped LiNi_0.5_Co_0.2_Mn_0.3_O_2_ Cathode Materials in 4.6 Voltage

**DOI:** 10.3389/fchem.2018.00643

**Published:** 2019-01-08

**Authors:** Zhaoyong Chen, Xiaolong Gong, Huali Zhu, Kaifeng Cao, Qiming Liu, Jun Liu, Lingjun Li, Junfei Duan

**Affiliations:** ^1^College of Materials Science and Engineering, Changsha University of Science and Technology, Changsha, China; ^2^College of Physics and Electronic Science, Changsha University of Science and Technology, Changsha, China; ^3^Department of Chemistry, University of New Hampshire, Durham, NH, United States

**Keywords:** lithium ion batteries, LiNi_0.5_Co_0.2_Mn_0.3_O_2_, co-doping, cation mixing, phase transition

## Abstract

The high energy density lithium ion batteries are being pursued because of their extensive application in electric vehicles with a large mileage and storage energy station with a long life. So, increasing the charge voltage becomes a strategy to improve the energy density. But it brings some harmful to the structural stability. In order to find the equilibrium between capacity and structure stability, the K and Cl co-doped LiNi_0.5_Co_0.2_Mn_0.3_O_2_ (NCM) cathode materials are designed based on defect theory, and prepared by solid state reaction. The structure is investigated by means of X-ray diffraction (XRD), rietveld refinements, scanning electron microscope (SEM), XPS, EDS mapping and transmission electron microscope (TEM). Electrochemical properties are measured through electrochemical impedance spectroscopy (EIS), cyclic voltammogram curves (CV), charge/discharge tests. The results of XRD, EDS mapping, and XPS show that K and Cl are successfully incorporated into the lattice of NCM cathode materials. Rietveld refinements along with TEM analysis manifest K and Cl co-doping can effectively reduce cation mixing and make the layered structure more complete. After 100 cycles at 1 C, the K and Cl co-doped NCM retains a more integrated layered structure compared to the pristine NCM. It indicates the co-doping can effectively strengthen the layer structure and suppress the phase transition to some degree during repeated charge and discharge process. Through CV curves, it can be found that K and Cl co-doping can weaken the electrode polarization and improve the electrochemical performance. Electrochemical tests show that the discharge capacity of Li_0.99_K_0.01_(Ni_0.5_Co_0.3_Mn_0.2_)O_1.99_Cl_0.01_ (KCl-NCM) are far higher than NCM at 5 C, and capacity retention reaches 78.1% after 100 cycles at 1 C. EIS measurement indicates that doping K and Cl contributes to the better lithium ion diffusion and the lower charge transfer resistance.

## Introduction

Nowadays, the vigorous development of lithium-ion batteries (LiBs) (Chen et al., 2017; Zhang et al., 2018a) has accelerated the production of energy storage devices (Zhang et al., 2018b; Zheng et al., 2018), electric vehicles (EVs), and hybrid electric vehicles (HEVs) (Terada et al., 2001; Goodenough and Park, 2013; Xiong et al., 2013, 2014b; Xu et al., 2015b; Choi and Aurbach, 2016; Liu et al., 2018b; Su et al., 2018). However, unsuitable performance limits the application of LiBs cathode materials, such as low energy density of LiCO_2_ and LiFeO_4_, and lithium-rich layered oxide (LRLO) cathode materials with low coulombic efficiency and voltage attenuation. Under these circumstances, researchers turn their attention to cathode materials with high energy density and low prices, therefore, lithium transition metal oxides (LiNi_*x*_Co_*y*_Mn_1−x−*y*_O_2_) due to its high capacity, low price (Chen et al., 2003; Shin et al., 2005; Li et al., 2009; Martha et al., 2009; Sun et al., 2009; Kim, 2013; Yue et al., 2013b; Xiong et al., 2014a) and its properties adjusted by the relative ratio of different TM ions (Kim et al., 2016) according to the requirement are diffusely researched, in particular, LiNi_0.5_Co_0.2_Mn_0.3_O_2_ (NCM) cathode materials has been attracting much more attentions.

It is all well-known that the Ni element plays a vital role in providing capacity for NCM. Unfortunately, the presence of Ni element also causes Ni to escape from the 3b sites into the 3a sites of the lithium layer during the preparation and charging because the radius of Ni^2+^ and Li^+^ is similar. And these defects are intensified during high-voltage cycling because of the increasing number of Li vacant sites. This Ni migration trigger cation mixing and phase transformation from layered (R-3m) to spinel (Fd-3m) and rock salt (Fm-3m) phase at some micro areas (Kojima et al., 2011; Boulineau et al., 2013; Jung et al., 2014; Lin et al., 2014), which results in structural degradation, poor cycle stability and slow lithium ion diffusion coefficient of NCM cathode material.

In the past few decades, extensive studies have been confirmed that ion substitution such as Na^+^ (Chen et al., 2013; Hua et al., 2014), Mg^2+^ (Luo et al., 2016), Fe^3+^ (Liu et al., 2006), Ti^4+^ (Seungtaek et al., 2005), V^5+^ (Zhu et al., 2014), F^−^ (Shin et al., 2006; Yue et al., 2013a) and so on is considered as an efficacious strategy to decrease the cation mixing degree, ameliorate the microstructure in stability and improve rate performance. Among them, Na^+^ substitution is regard as a typically dopant to ameliorate the performance of NCM. Li_1.1−x_Na_*x*_Ni_0.2_Co_0.3_Mn_0.4_O_2_ (Park et al., 2006) are prepared by sol-gel method with better rate performance, and lower cation mixing are exhibited when x was 0.05 and 0.1. But he cycle stability and structural stability of the material have not been apparently improved. In addition, many researchers further improve the stability of the material during cycling and enhance the electrochemical performance of the material by anionic doping. For instance, G-H. Kim et al. (Kim et al., 2005) synthesized LiNi_1/3_Co_1/3_Mn_1/3_O_2−z_F_*z*_ by partially replacing O with F, and improves structural stability of materials. However, it did not solve the cation mixing and improve the rate performance.

As far as we know most of these attainable studies are limited to a single replacement and do not synchronously improve the cycle stability, lithium ion diffusion coefficient and cation mixing. Therefore, in this study, aiming to improving the structure stability and rate performance under 4.6 V, we designed K and Cl co-doped Li_0.99_K_0.01_Ni_0.5_Co_0.2_Mn_0.3_O_1.99_Cl_0.01_ (KCl-NCM) cathode material and prepared it using solid-state reaction. Because of the tangible that the radius of K^+^ (${\text{r}}_{\text{K}}^{+}$ = 1.33 Å) is much larger than that of Li^+^ (${\text{r}}_{\text{Li}}^{+}$ = 0.76 Å), we partially replace Li with K into the structure of NCM to reduce the mixing of the cations and improve the lithium ion diffusion coefficient. Simultaneously, we also partially replace O with Cl into the crystal structure because of the covalent radii and the electronegativity of Cl much than O (Singh et al., 2017), moreover, Cl doping is associated with the reinforcement of MnO_6_ octahedral in the framework by the strong ionic Mn-Cl, Ni-Cl, and Co-Cl bonds (Kim et al., 2014), which makes the structure more stable and improves cyclic performance. Through the co-doping, cycle performance and rate performance of NCM are markedly improved. Moreover, the content of Ni occupies Li sites (2.77%) for the KCl-NCM is lower than NCM (3.3%) identified by Rietveld refinements, which effectively reduces the cation mixing.

## Experimental

### Preparation of the Samples

Li_0.99_K_0.01_Ni_0.5_Co_0.3_Mn_0.2_O_1.99_Cl_0.01_ (KCl-NCM) layered cathode materials were prepared via solid-state reaction using stoichiometric of KCl, commercial transition-metal hydroxide precursors Ni_0.5_Co_0.2_Mn_0.3_(OH)_2_ and LiOH·H_2_O as raw materials, wherein the ratio of Li to the transition metal is 1:1, K and Cl were added to the mass fraction of 1%. The raw materials were mixed at an agate mortar, and grind time was 1 h to make it fully mixed, then which was heated at 480°C for 2 h and calcined at 880°C for 12 h at a heating rate of 5°C min^−1^ in air. Finally, the sample was cooled slowly in the furnace to room temperature. Meanwhile, synthesis conditions of LiNi_0.5_Co_0.3_Mn_0.2_O_2_ are consistent with KCl-NCM except that a certain stoichiometric ratio of KCl is added, which is regard as reference sample.

### Materials Characterization

X-ray diffraction (XRD, Rigaku D/Max 200PC, Japan) analysis was carried out on a Rigaku/Max-RAX powder diffractometer with Cu Kα-radiation. The scanning speed is 5° min^−1^ and scanning range is 10° < 2θ < 90°. The morphologies and microstructures of all samples were determined by scanning electron microscopy (SEM, Nova NanoSEM-230), and energy dispersive X-ray spectroscopy (EDS) is carried out on OXFORD7426 as the attachment of SEM, with the acceleration voltage of 20 kV. Transmission electron micrographs (TEM) were recorded by a JEOL JEM-2010 transmission electron microscope.

### Electrochemical Measurement

The positive electrode (about 4.30 mg cm^−2^) consists of 80 wt.% as-prepared composites, 15 wt.% acetylene black and 5 wt.% polyvinylidene fluoride (PVDF) as a binder, and metal Al foil is used as collector. Celgard 2,400 is used as separator which is soaked in 1.0 mol L^−1^ LiPF_6_/EC+DMC (EC:DMC = 1:1 in volume ratio) electrolyte. Lithium metal foil is used as the counter electrode during electrochemical measurements. All the cells are assembled in an argon-filled glove box. The charge/discharge test is carried out by using a Land BT2001A automatic battery test system in the voltage range of 2.7~4.6 V, and the density of current is measured by 1 C (1 C means 150 mAh g^−1^). The electrochemical impedance is measured in the frequency range from 10^−3^ to 10^5^ Hz on a CHI660B electrochemical working station (Chenhua, Shanghai, China), and the perturbation amplitude is controlled at ±5 mV.

## Results and Discussion

### Structural Characterization

**Figure 1:**
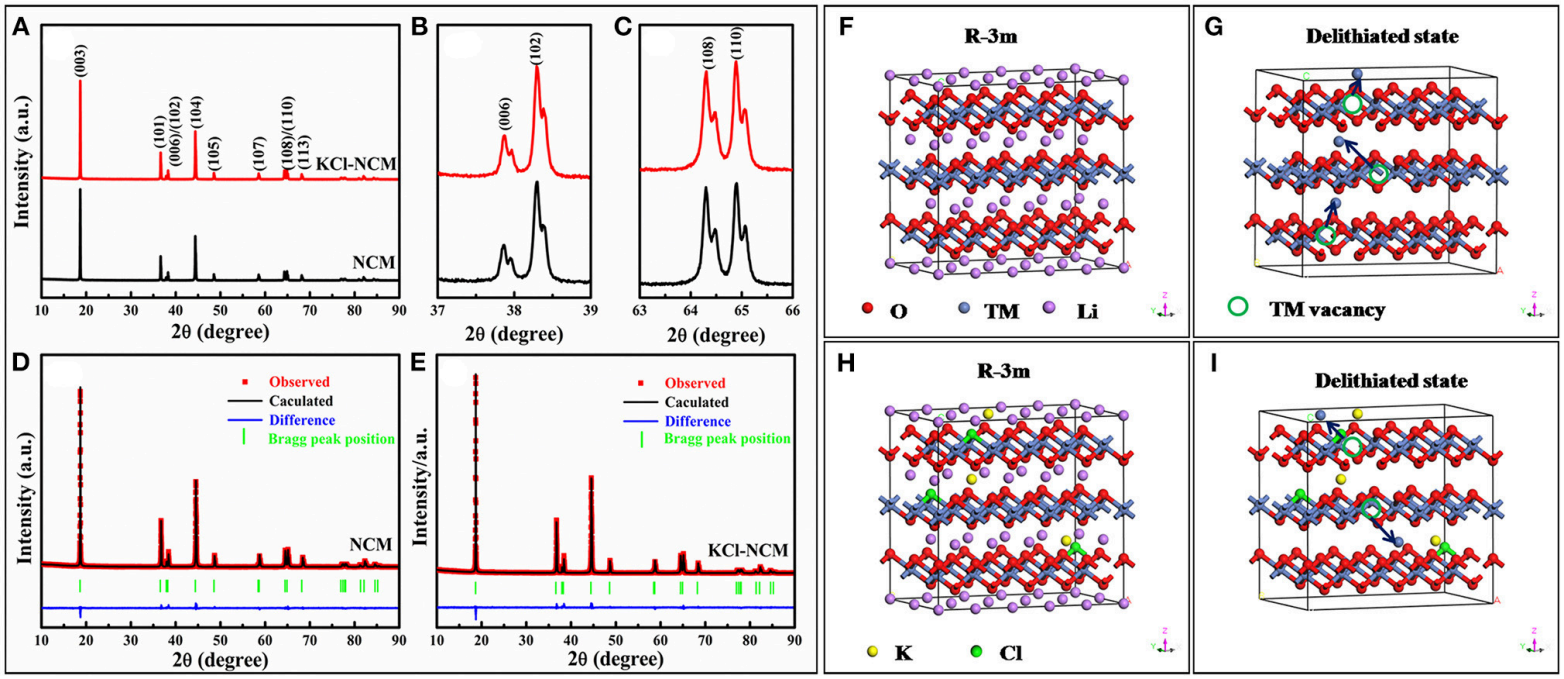
**(A)** XRD patterns of NCM and KCl-NCM samples, **(B)** and **(C)** are partial enlarged views of **(A)**; Refinements patterns for samples: **(D)** NCM **(E)** KCl-NCM; well-ordered R-3m structure of samples: **(F)** NCM **(G)** KCl-NCM; partially cation mixed phase with TM ions in Li slab at highly charged state: **(H)** NCM **(I)** KCl-NCM.

Figure 1A displays the XRD patterns of NCM and KCl-NCM. From XRD patterns, we can observe that all the samples are indexed to a R-3m structure of hexagonal, and no other impurities is detected. From the Figures 1B,C, we can clearly observe that the peaks of (006)/(102) and (108)/(110) are separated, indicating that the material have a good layered phase structure (Lee et al., 2013; Zhu et al., 2014; Xu et al., 2015a). The lattice constants c/a and R(I_003_/I_104_) of all samples are shown in Table 1. When K and Cl are co-doped into the NCM crystals, the lattice constants increase obviously, indicating that K and Cl are successfully incorporated into the crystal lattice. It was reported that the R value of the samples is >1.2, and also increases after doping, which indicates the cation mixing is reduced to a certain degree. It will be beneficial to the improvement of the electrochemical properties of the material.

**Table 1:** Lattice constants of NCM and KCl-NCM samples.

**Sample**	**a (Å)**	**c (Å)**	**c/a**	**R(I_**003**_/I_**104**_)**	**V (Å^**3**^)**
NCM	2.86735	14.21039	4.956	1.319	101.18
KCl-NCM	2.87407	14.26912	4.965	1.497	101.58

To further explain the role of K substitution for Li in the Li layers, rietveld refinements is used to further analyze the XRD pattern of the samples (Li et al., 2012b). It is assumed that Li, TM, and O occupy the 3a, 3b, and 6c sites, respectively (Chen et al., 2013). In this work, we assume that K completely occupies the Li site, which leads to the highest reliability factors. And the pictures of Rietveld refinements are shown in Figures 1D,E. Table 2 is occupancies of atoms for all samples. Obviously, it can be seen that the Ni/Li mixing degree is decreased prominently by K substitution. Furthermore, compared with NCM (3.3%), the Ni content in the Li layer (2.77%) of KCl-NCM is lower. The result can be attributed to the incorporation of K^+^ into the Li layer, which would generate a big driving force to separate Li^+^ ions from the transition metal layer and thus avoid the Li/Ni disorder of the KCl-NCM. Hence, the substitution of Li^+^ by K^+^ leads to a more ordered layered structure, a larger Li layer distance, and a lower cation mixing degree in KCl-NCM. In order to make the results of Rietveld refinements and XRD more specific, we simulate the cation disorder with R-3m structure for NCM and KCl-NCM. Figures 1F,H present a perfect R-3m structure of Li-oxygen-TM-oxygen-Li, which clearly separates TM sites (3b) and lithium sites (3a). But Ni ions are easy to enter into the Li layers because the similar to the ionic radius of Ni^2+^ and Li^+^ during the highly charged state, as shown Figure 1G. Figure 1I shows TM ions in Li slab at highly charged state for KCl-NCM, since the K^+^ radius is much larger than the radius of Ni^2+^, which reduces the number of Ni^2+^ migration to the Li site. As a result, K^+^ doping can bring down the cation mixing to some extent, and it is also consistent with the results of the Rietveld refinements.

**Table 2:** The results of Rietveld refinements for NCM and KCl-NCM samples.

**Atom**	**site**	**x**	**y**	**z**	**NCM**	**KCl-NCM**
Li1	3a	0	0	0	0.9663	0.9619
Ni2	3a	0	0	0	0.0337	0.0277
Ni1	3b	0	0	0.5	0.4663	0.4723
Co1	3b	0	0	0.5	0.2	0.2
Mn1	3b	0	0	0.5	0.3	0.3
K1	3a	0	0	0	0	0.0104
O1	6c	0	0	0.2411 (1)	2	1.99
Cl1	6c	0	0	0.2411 (1)	0	0.01
Rwp	–	–	–	–	4.36%	4.33%

**Figure 2:**
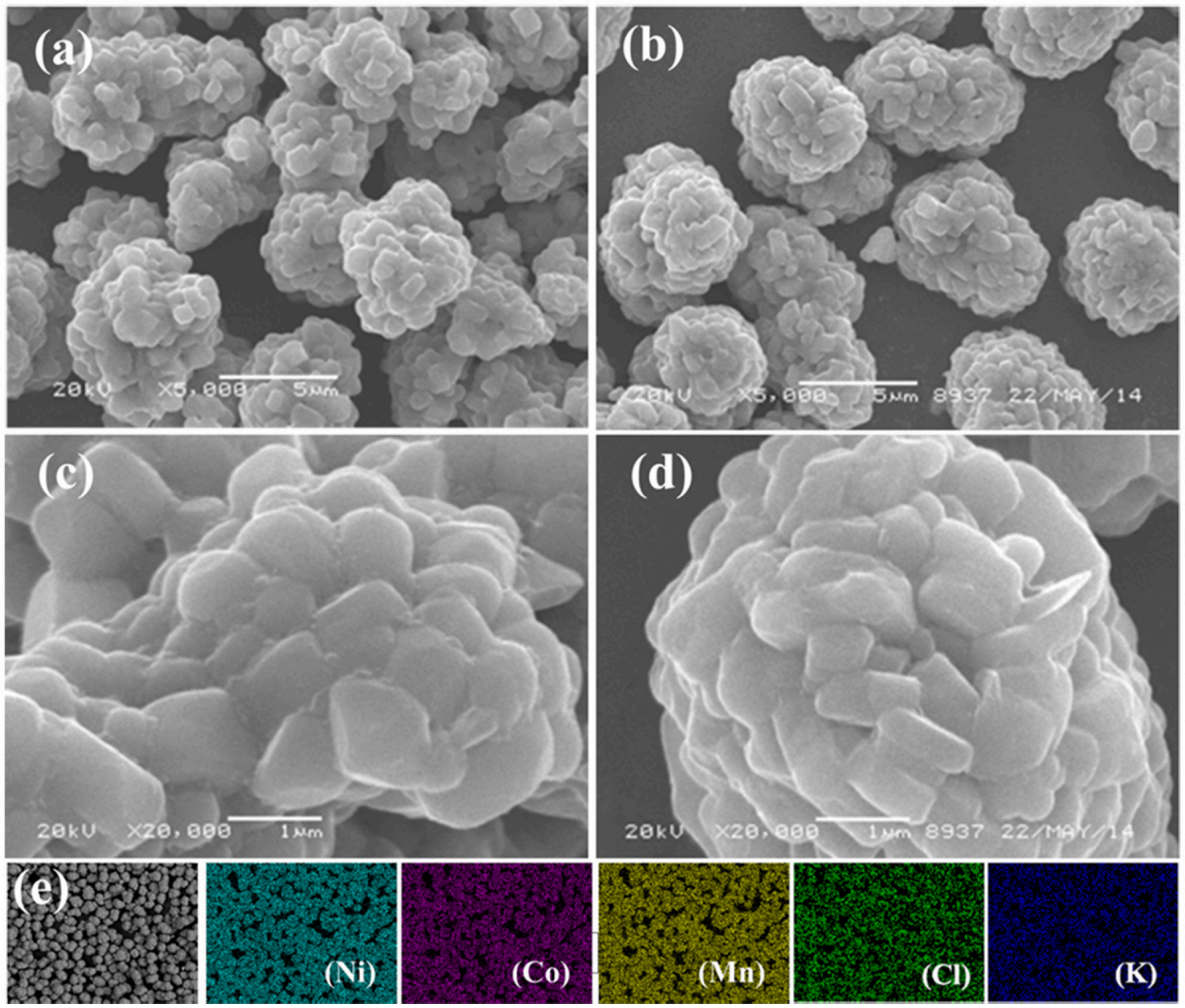
SEM images of NCM **(a,c)** and KCl-NCM **(b,d)**; EDS mappings of KCl-NCM **(e)**.

The SEM images of NCM, KCl-NCM and the corresponding EDS mappings are illustrated in Figure 2. A uniform near-spherical microstructure of about 5 microns can be observed, which are agglomerated by uniform size of a particle. The corresponding EDS mappings of KCl-NCM display all elements including K and Cl are uniformly distributed, which reveals K and Cl are successfully incorporated into the NCM.

**Figure 3:**
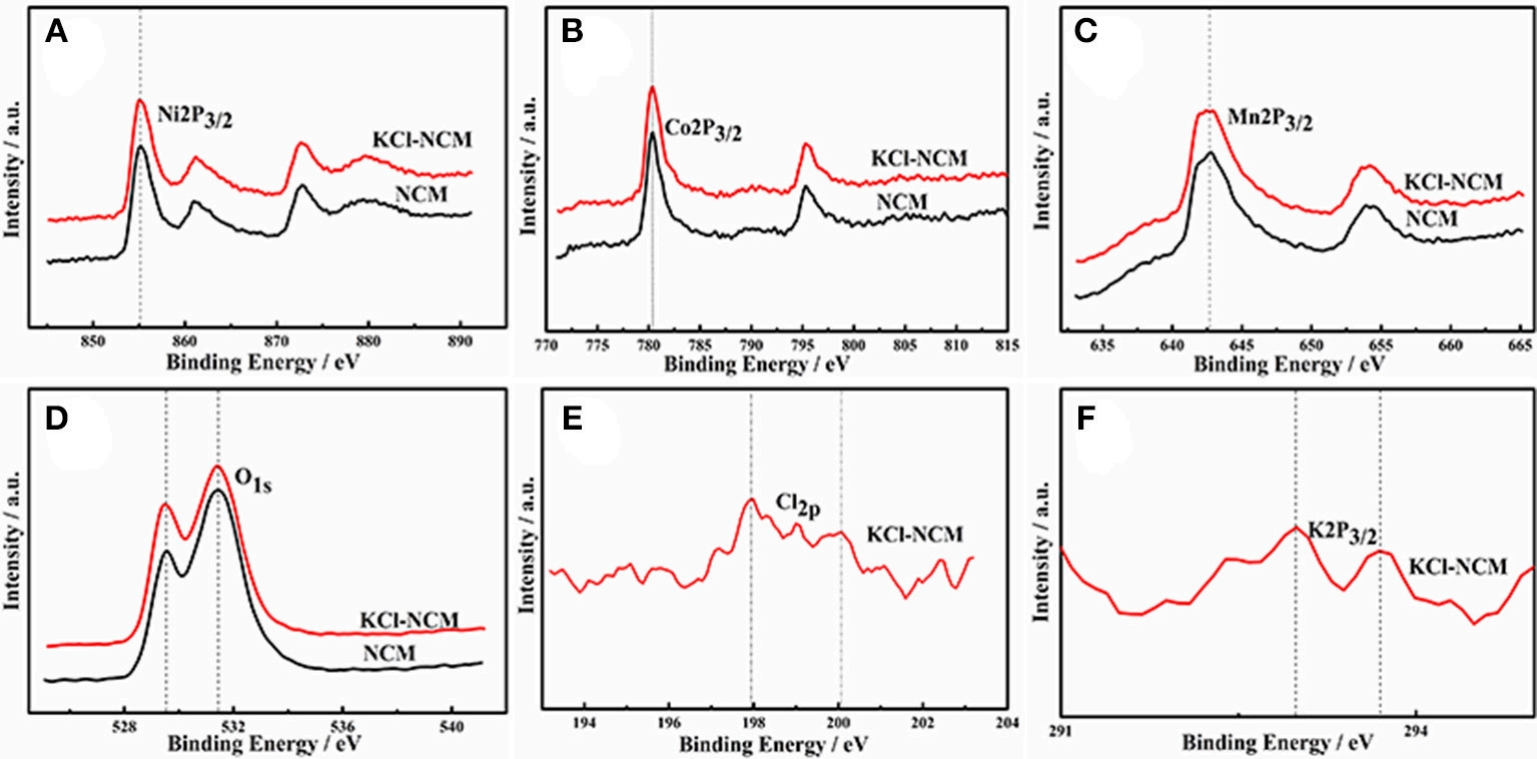
XPS images of NCM and NCM-KCl. **(A)** Ni2p_3/2_; **(B)** Co2p_3/2_; **(C)** Mn2p_3/2_; **(D)** O_1s_; **(E)** Cl_2p_; **(F)** K2P_3/2_.

To further determine the signal of K and Cl, XPS is performed. Figure 3 shows the XPS patterns of transition metal elements Ni, Co, Mn, K, Cl and O in LiNi_0.5_Co_0.3_Mn_0.2_O_2_ samples before and after KCl doping, as shown, the electron binding energies of Ni2p, Co2p, and Mn2p in LiNi_0.5_Co_0.3_Mn_0.2_O_2_ samples obtained by doping with KCl have not change significantly, which are 855.3 eV, 780.4 eV and 642.8 eV, respectively, the observed binding energies for Ni 2p_3/2_, Co 2p_3/2_ and Mn2p_3/2_ of oxidation state coincide well. The binding kinetics peaks of K and Cl are shown in samples doped with KCl, indicating that the dopant elements are present in the sample.

**Figure 4:**
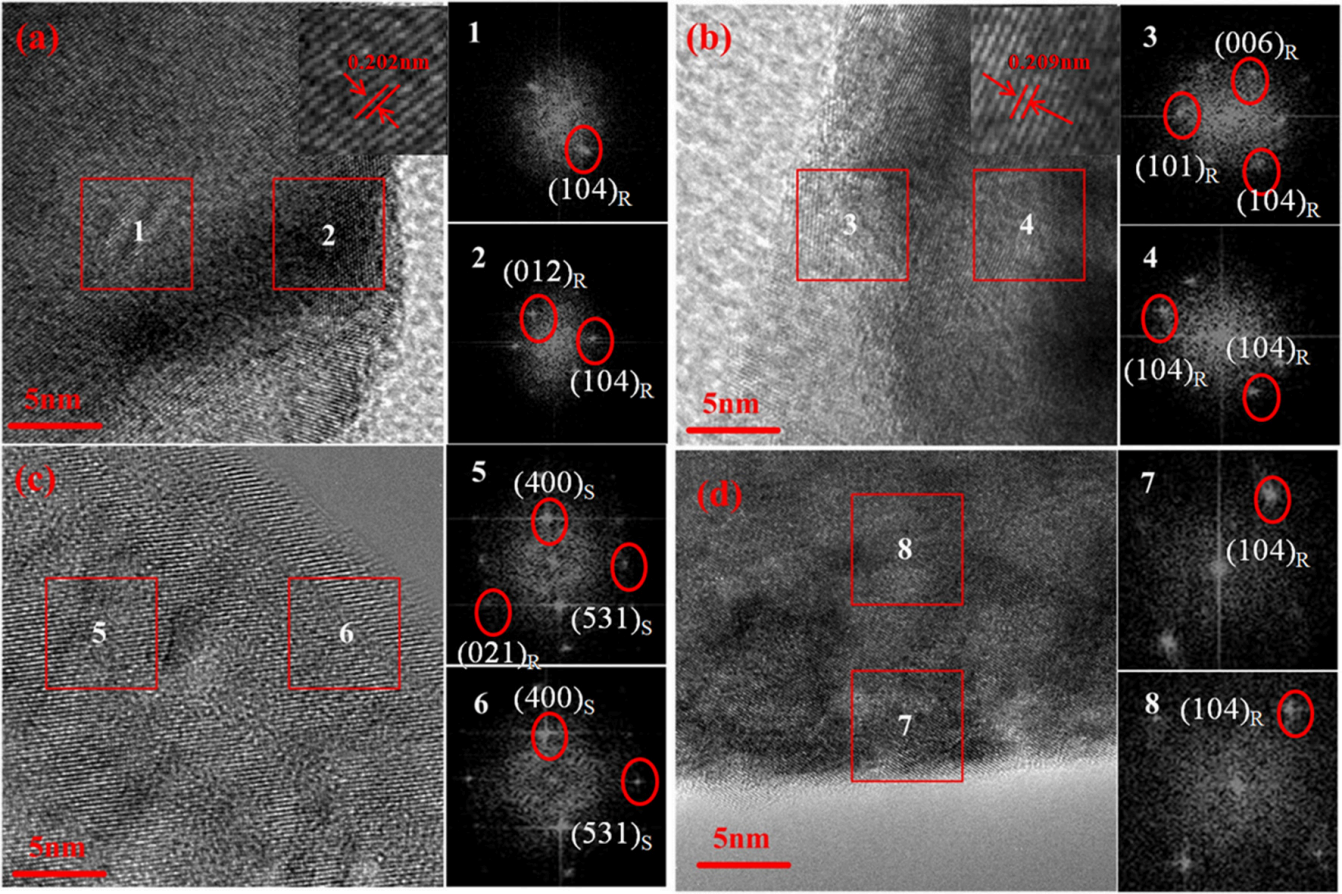
HRTEM images and the corresponding Fast Fourier Transform (FFT) patterns of **(a)** NCM, **(b)** KCl-NCM before cycling, and **(c)** NCM, **(d)** KCl-NCM after 100 cycles.

To provide the detailed information and investigate local structural changes of the samples, high-resolution transmission electron microscopy (HRTEM) and fast fourier transformation (FFT) are conducted on NCM and KCl-NCM. Various regions in the sample are examined to avoid any confusion. Figures 4a,b exhibit a good layered structure and no any trace of a secondary phase regardless of near the surface or the inner region before electrochemical testing, which reveals that K and Cl co-doping have not destroy the layered structure of NCM. Moreover, from the insets in Figures 4a,b, we can clearly see that the interplanar spacing of the sample doped with K^+^ and Cl^−^ is larger than NCM sample, indicating that the doping of K^+^ enlarges the spacing of Li layers, which is consistent with the result that the c value of the KCl-NCM sample is larger than the c value of the NCM sample in the XRD. As a result, it will also contribute to improve the rate performance. However, the local structure has change dramatically after cycling 100 times at 4.6 V for NCM (Figure 4c). The additional crystal planes can be indexed as (400)_S_ and (531)_S_ in Figure 4c compared with Figure 4a, corresponding to a spinel structure. It indicates that NCM undergoes a transition from hexagonal phase to spinel phase in cyclic testing. In general, Ni ions occupying Li sites will lead to Li deficiency, and it can give rise to phase transformation at some micro areas. And it triggers the collapse of the layered structure. In contrast, we find that the structure of K and Cl co-coped sample (Figure 4d) is distinctly different from that of the NCM sample after 100 cycles at 4.6 V. A well-layered structure is still maintained after high-voltage cycling, corresponding to the (104)_R_ of the FFT images. This enhanced structural stability is attributed to the K substitution, which reduces the mixing of Li and Ni, suppressing it from the severe structural degradation induced during charge and discharge process. As a result, this suppression of phase transition intensely ameliorates the deterioration of electrochemical performance of Ni-rich cathode materials during high-voltage cycling (Yang and Xia, 2015).

### Electrochemical Performance

**Figure 5:**
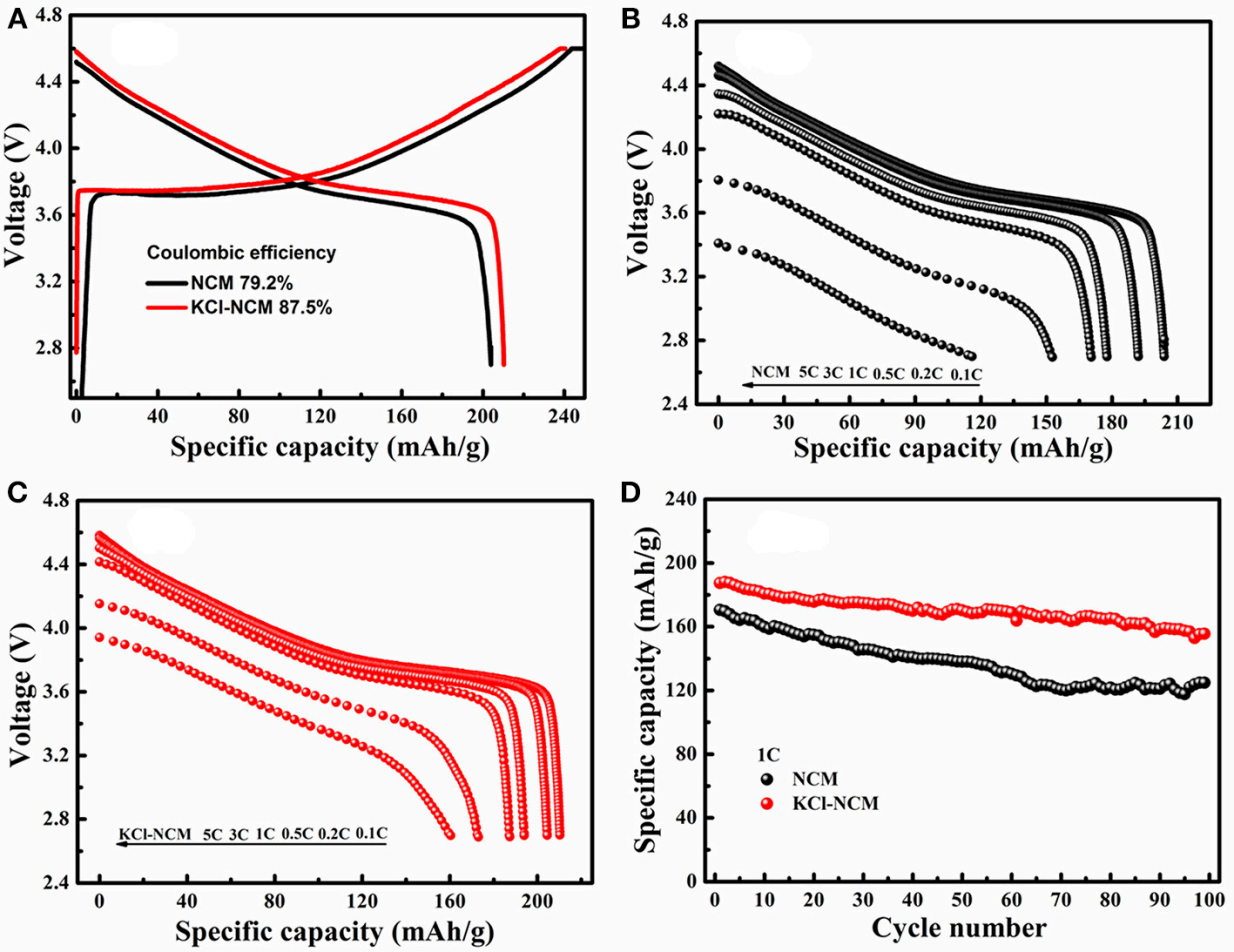
Electrochemical performance: **(A)** First charge/discharge profile for the NCM and KCl-NCM at a rate of 0.1 C; discharge profile of **(B)** NCM, **(C)** KCl-NCM at different rate; **(D)** cycle performance at 1 C for NCM and KCl-NCM.

Figure 5 describes electrochemical performance of NCM and KCl-NCM. Figure 5A illustrates atypical initial charge-discharge curve of the NCM. The initial discharge capacity for the NCM and KCl-NCM is 203.9 and 210.3 mAh/g. In contrast, it is obvious that the coulombic efficiency and initial discharge capacity of KCl-NCM sample is superior to those of NCM. The rate capacity of NCM and KCl-NCM is evaluated in Figures 5B,C, the discharge capacity of NCM samples drops dramatically with the current density increasing, and the discharge capacities of NCM are from 203.9 mAh g^−1^ at 0.1C to 152.74 and 116.0 mAh g^−1^ at 3 C and 5 C, which are only 74.9 and 56.9% of the discharge capacity at 0.1 C. However, the discharge capacities of the sample doped with K and Cl at 3 C and 5 C is, respectively, 175 and 162.5 mAh/g, corresponding to 83.7 and 77.7% of its capacity of 209.1 mAh/g at 0.1 C. Apparently, the rate performance of K and Cl substituted sample is remarkably enhanced compare with NCM, which may be due to the fact that K replaces the Li site and increases the diffusion channel of lithium ions because the radius of K^+^ (${\text{r}}_{\text{K}}^{+}$ = 1.33 Å) is higher than that of Li^+^ (${\text{r}}_{\text{Li}}^{+}$ = 0.76 Å), in addition, according to the literature (Singh et al., 2017), the doping of Cl plays a role in the improvement of the rate performance because the radius of Cl is larger than the radius of O. Figure 5D demonstrates the cycle performance of two samples at 1 C rate. The remaining discharge capacity for NCM after 100 cycles is 124.8 mAh/g, and the capacity retention is 73.2%. With regard to KCl-NCM, the discharge capacity is 155.54 mAh/g after 100 cycles, and the capacity retention is improved to 83.0%. The cycle performance of sample co-doped with K and Cl is significantly improved. The possible reason is the fact that K substitution reduces the mixing of Li and Ni. On the other hand, Cl substitution can reduce the reactivity of the cathode toward electrolyte oxidation and associate with the reinforcement of MnO_6_ octahedral in the framework by the strong ionic Mn-Cl, Ni-Cl, and Co-Cl bonds (Kim et al., 2014). Therefore, K and Cl substitution synergistically improved the rate performances and the structure stability during cycling.

**Figure 6:**
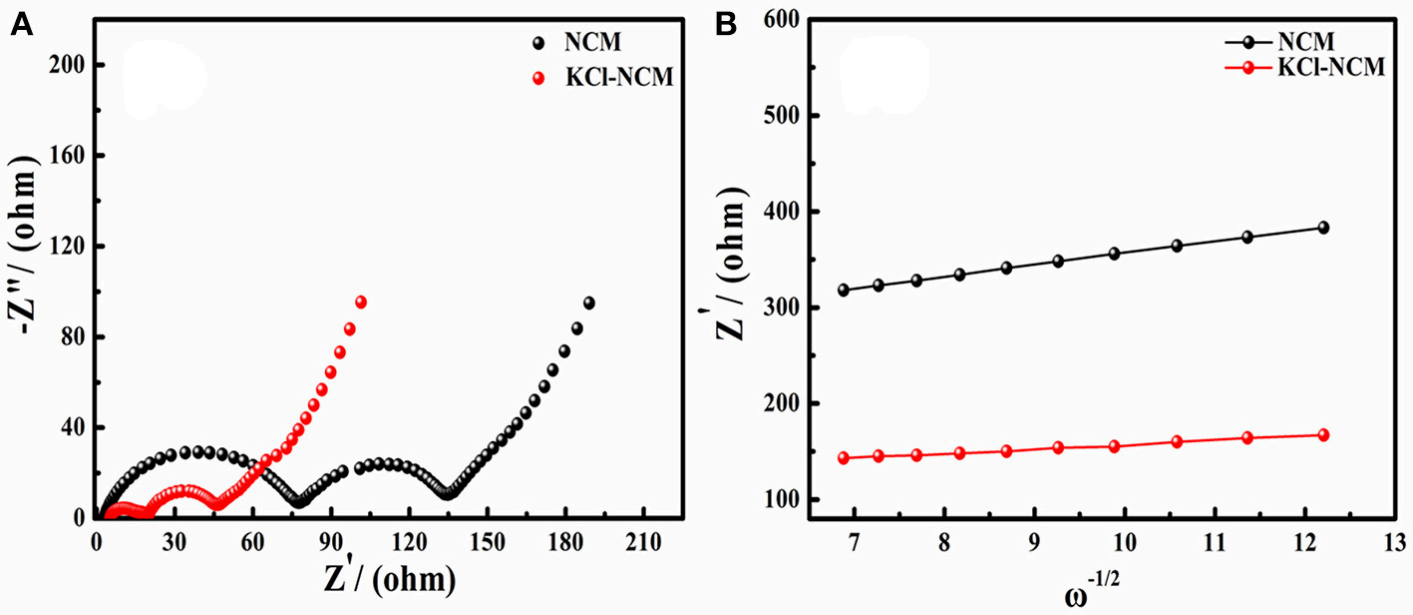
**(A)** EIS plots of NCM and KCl-NCM; **(B)** corresponding to the relationships between $Z_{\text{re}}^{\prime }$ and ω^−1/2^.

To further understand the effect of K and Cl doping on the lithium ion transport of NCM cathode materials, the electrochemical impedance spectroscopy (EIS) and corresponding relationships between $Z_{\text{re}}^{\prime }$ and ω^−1/2^ conducted are shown in Figure 6. The diffusion coefficient of lithium ion (D_Li+_) can be calculated via the equation as described in references (Li et al., 2012a; Mai et al., 2013; Zheng et al., 2014, 2019; Choi et al., 2015; Liu et al., 2018a). From the Figure 6 and Table 3, we can see that the impedance of NCM and KCl-NCM samples are 134.8 and 46.4 Ω, and it is clear that the doping K and Cl reduces the electrode resistance of the sample. Compared to the undoped sample, the diffusion coefficients of lithium ions doped with K and Cl increases from 2.62 × 10^−10^ to 2.37 × 10^−9^cm^2^ s^−1^. Generally, the D_Li+_ is known as an intrinsic property for a given positive electrode, which depends only on the structure of active material in the charge state. It has been proven that the activity energy for the Li-ion transport in solid could be reduced effectively for the reason of increasing Li layer distance and reducing cation mixing (Hua et al., 2014). So, the doped samples can offer a large amount of lithium ion in the intercalation and deintercalation reaction at large charge and discharge current. Therefore, KCl-NCM have a faster Li diffusion probably due to the larger Li layer spacing and the lower Li/Ni disorder. The decrease of the impedance and the increase of the diffusion coefficient of the lithium ion show that the KCl-NCM reduce the polarization of the electrode, and improves the cycle performance, which is consistent with the electrochemical test result.

**Table 3:** The values of R_s_ + R_ct_ and D_Li__+_ for NCM and KCl-NCM.

**Samples**	**R_**s**_ + R_**ct**_ (ohm)**	**D_**Li+**_ (cm^**2**^s^**−1**^)**
NCM	134.8	2.62 × 10^−10^
KCl-NCM	46.4	2.37 × 10^−9^

**Figure 7:**
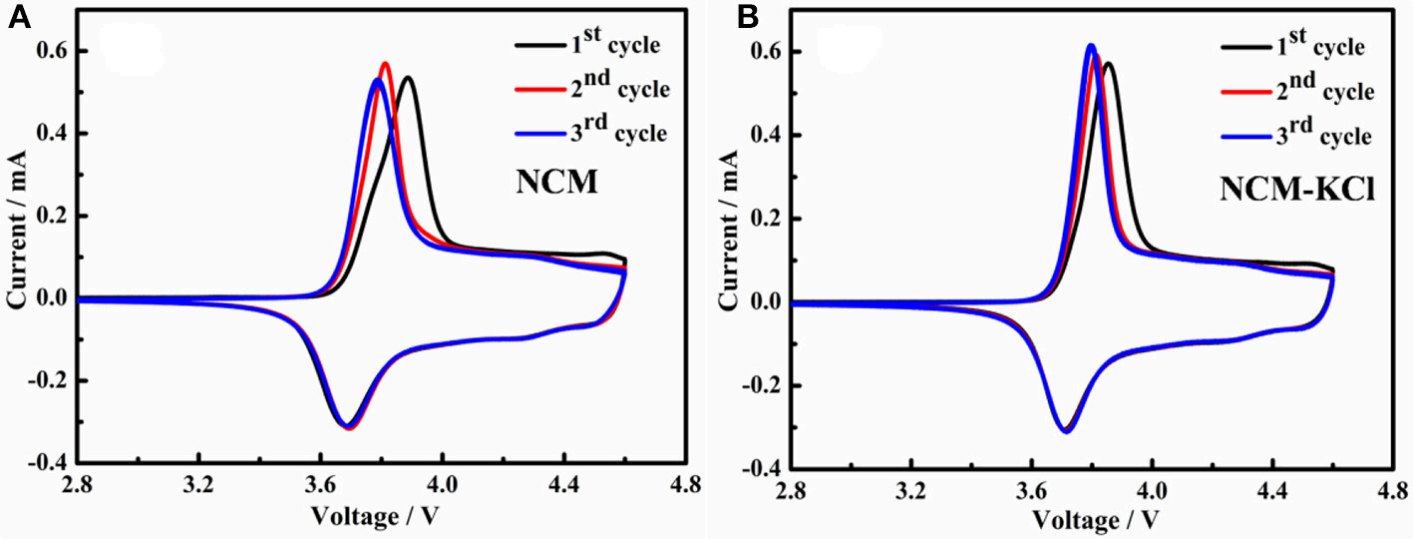
Cyclic voltammogram of samples cells at the scan rate of 0.1 mV s^−1^: **(A)** NCM; **(B)** NCM-KCl.

Figure 7 presents the cyclic voltammogram of two samples. As can be seen from Figure 7, these CVs demonstrate quite reversible electrochemical behavior with well resolved oxidation/reduction peaks related to the Li-extraction/insertion accompanied with the Ni^2+^/Ni^4+^ and Co^3+^/Co^4+^ oxidation/reduction, respectively. From the Table 4, the oxidation peaks for NCM and NCM-KCl of the first cycle centerat 3.8873 V and 3.8598 V, corresponding to the reduction peaks centerat 3.6827 V and 3.7131 V, respectively, it is obviously that the difference value between the oxidation peaks and reduction peaks for the KCl-NCM (0.1461 V) is smaller compared to NCM (0.2046 V), and the same pattern is presented in the second and third cycle. It is well-known that the bigger the potential difference between lithium ions intercalating and deintercalating, the stronger the electrode polarization is. This smaller difference between oxidation and reduction peaks positions indicates the better reversibility of Li^+^ ions during intercalating/deintercalating in the KCl-NCM materials, which is consistent with the result of initial charge-discharge curves for the NCM and KCl-NCM. Meanwhile, it ensures reduced capacity fade during cycling. Therefore, K and Cl co-doped can weaken the electrode polarization and improve the electrochemical performance.

**Table 4:** The results of cyclic voltammogram for NCM and NCM-KCl.

**Samples**	**Oxidation peaks (V)**	**Reduction peaks(V)**	**Difference value (V)**
**NCM**			
1st cycle	3.8873	3.6827	0.2046
2nd cycle	3.8115	3.6954	0.1161
3rd cycle	3.7871	3.6907	0.0964
**NCM-KCl**			
1st cycle	3.8598	3.7137	0.1461
2nd cycle	3.8076	3.7145	0.0931
3rd cycle	3.7854	3.7164	0.069

## Conclusion

In a word, we have researched out an effectual method to improve the structural stability and electrochemical performance of the Ni-rich layered oxide cathode during high-voltage cycling. By XRD and TEM analysis, it is found that the dopant materials have a higher cation ordering degree and complete layered structure. Rietveld refinements prove K and Cl substitutes can effectively reduce cation mixing. Through electrochemical performance analysis, KCl-NCM has a better comprehensive performance compared to NCM. The initial capacity is improved, at the same time the rate performance has also been greatly improved because of reducing the electrode impedance and improving lithium ion diffusion coefficient. Especially, doping K and Cl into the layered structure of NCM could effectually inhibit the phase transition to some degree during high-voltage cycling, leading that layered structure of KCl-NCM remains more complete than NCM after 100 cycles.

## Author Contributions

ZC and XG conceived the idea. XG and ZC prepared all materials and wrote the manuscript. HZ, KC, and XG analyzed the data. QL and JL conducted XRD, SEM, and TEM experiments. JD and LL played active roles in providing constructive suggestions.

### Conflict of Interest Statement

The authors declare that the research was conducted in the absence of any commercial or financial relationships that could be construed as a potential conflict of interest.
